# In vivo terahertz sensing of psoriasis and eczema patients

**DOI:** 10.1038/s41598-024-68106-2

**Published:** 2024-07-30

**Authors:** Jacob J. Young, Arturo I. Hernandez-Serrano, Joesph Hardwicke, Emma Pickwell-MacPherson

**Affiliations:** 1 Department of Physics, University of Warwick, Coventry, CV4 7AL UK; 2 Institute of Applied and Translational Technologies in Surgery (IATTS), University Hospital Coventry and Warwickshire, Coventry, CV2 2DX UK; 3 Medical School, University of Warwick, Coventry, CV4 7AL UK

**Keywords:** Mid-infrared photonics, Skin diseases

## Abstract

In this study we present the first in vivo clinical study of patients with eczema and psoriasis using terahertz (THz) sensing. Eczema and psoriasis patients were measured using a handheld THz scanner, both before and after the application of moisturiser. We show that THz sensing can distinguish between dry and healthy skin in different regions of the body. Furthermore, the impact of applying moisturiser on the skin can also be observed and potentially evaluated using THz light.

## Introduction

**Figure 1:**
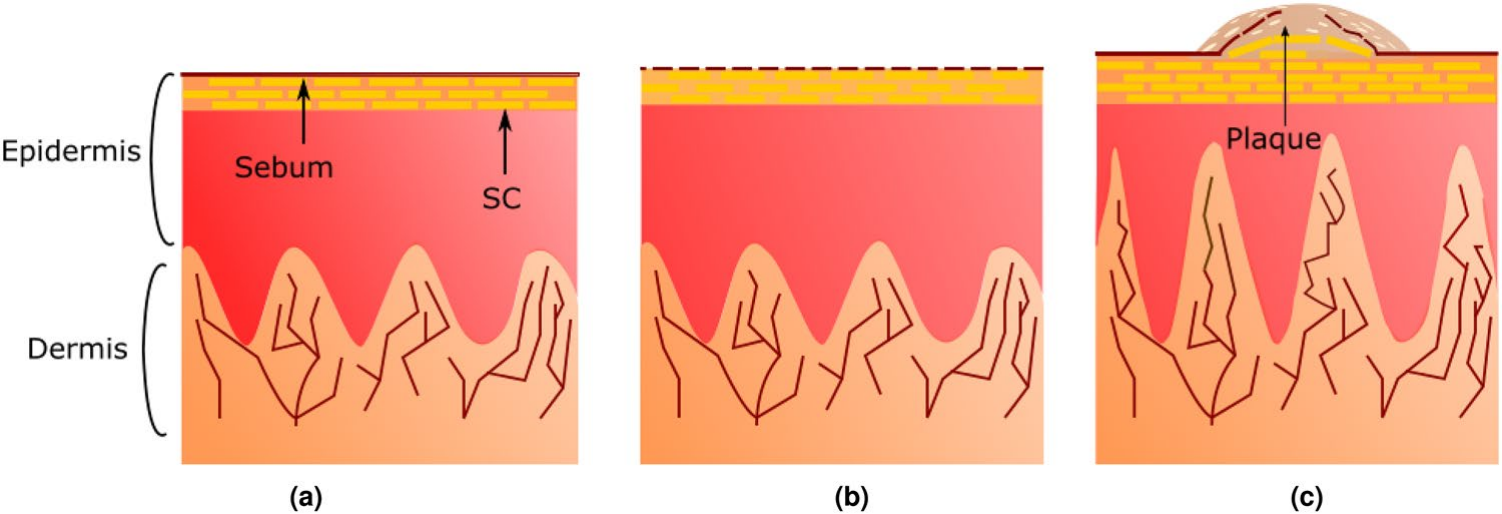
Healthy skin (**a**) consists of the dermis and epidermis. The epidermis is made up of the stratum corneum (SC) with a thin sebum layer on top. Eczematic skin (**b**) has a cracked sebum layer, leading to a drier epidermis and allowing the ingress of allergens and dirt. Psoriatic skin (**c**) features swollen blood vessels in the dermis and a cracked plaque on the surface of the SC.

Skin is the largest organ on the human body and acts as a protective barrier. As illustrated in Fig. 1a, it is a layered structure comprising (from outermost to innermost) the epidermis, dermis and subcutaneous tissue. The outermost layer of the epidermis is the stratum corneum (SC) and on top of this there is a thin sebum layer. The cells forming the SC are sloughed off in the skin cycle - they are the oldest cells and are drier than the cells in the remaining epidermis beneath them. For clarity, in this paper hereon we will refer to the remaining epidermis beneath the SC as simply the epidermis. Eczema is a common dry skin condition that affects 4 % of the UK population^1^. Typically, this condition causes the sebum layer to become cracked (Fig. 1b), this then allows allergens and dirt to enter the SC which in turn can lead to further irritation of the skin causing itchiness and further damage. Depending on the severity this can lead to red and blistered skin.

Psoriasis is another common condition affecting 2-3 % of the UK population^2^. Psoriasis is an immune-mediated condition, which can cause symptoms on the skin and the joints. For those suffering with psoriasis on the skin, the skin replacement process is sped up, such that the skin cycle, which would usually take 21-28 days, takes just a few days to replace skin cells. This causes skin cells to accumulate and form raised ‘plaques’ on the skin, which can also be flaky, scaly and itchy. They may become red on Caucasian skin and form darker patches on darker skin tones. Figure 1c illustrates the formation of a psoriasis plaque.

Dry skin conditions such as eczema and psoriasis are often diagnosed by eye and treatment success is assessed based on patient reports and visual inspection. Methods such as Raman spectroscopy can be used to show the hydration profile^3^ of dry skin however this is impractical as these devices are large, expensive and they are unsuitable for measuring small areas such as behind the ear or the side of the nose. This means there is a need for a portable, cheap and fast method to both diagnose and monitor the treatment of dry skin conditions: here we investigate the potential of terahertz (THz) sensing to do this.

THz light is non-ionising and would therefore be safe to use to scan patients as part of a screening or monitoring programme. Furthermore, it is highly sensitive to water content and, as such has the potential to be able to detect subtle changes in hydration of biological tissues such as the skin^4–6^, cornea^7^ and other epithelial linings including the colon^8^. Early studies of excised tissues have shown that the optical properties of tissues with cancer are different from their healthy counterparts. Ashworth *et al*^9^ report the frequency dependent properties of breast cancer for up to 2 THz and show statistically significant differences in the complex refractive index. Similarly, excised basal cell carcinoma was found to have a higher refractive index than healthy skin^10^.

Taylor *et al.* have used stratified medium theory to model the water concentration gradient across the cornea of the eye. In this approach the cornea is modelled as multiple thin layers with different hydration values^11^. We have applied stratified medium theory to model skin in our study measuring 300 volunteers^12^ and shown how THz sensing can be used to quantify changes in water content and thickness of the SC. Some of the volunteers in the study reported that they suffered from dry skin and their THz data typically resulted in a lower SC hydration value, however other factors such as exercise and application of moisturiser can also affect the results^13^.

Previous studies have shown that THz sensing could be used to predict healing outcomes in burns of porcine skin models^14^ and can also probe the hydration of skin through transdermal patches^15^. Recent work by Qi *et al*^16^ shows the use of a non-contact QCL narrow band THz system for the in vivo imaging of various skin pathologies and cancers. However, this was done in a laboratory environment whereby patient volunteers were brought to the THz system at the university. Hitherto, a clinical in vivo study has not been carried out. Herein, we report the first time that THz equipment has been taken to a hospital to measure dry skin patients. We focus on psoriasis and eczema patients as we have measured 38 such cases (24 psoriasis and 14 eczema).

## Results

A total of 38 dry skin patients were measured as part of the SINATRA study: SkIN hydrAtion evaluation with TeRAhertz scanning. The break down of the number of patients measured with different conditions is summarised in table 1. THz measurements were made continuously for 60 seconds using the handheld reflection set up at a rate of 4 Hz. The region of interest (ROI) for each patient was aligned to the THz optics using a quartz imaging window pressed to the skin surface.

**Table 1:** Number of patients measured as part of the SINATRA study, categorised by skin condition.

**Condition**	**No. measured**	**No. of photos**
Eczema	14	5
Psoriasis	24	3
Total	38	8

The fundamental changes in the optical properties of skin in the frequency domain cause differences to also be seen in the reflected THz signals that are measured in the time domain. The time domain signal also gives information about the structure of the skin - different layers, such as the SC, will cause reflections which can be analysed to deduce the hydration and thickness as a function of depth^12^. The MacPherson group has done numerous skin studies over the past two decades and typically uses the impulse response function to analyse the data initially as this removes any system fluctuations and provides a method to make direct comparisons between measurements of different subjects taken on different days. The impulse response function also enables us to understand the sample structure, which in turn affects the subsequent assumptions and calculations we can make. Due to the multiple layers in the dry skin, it is not meaningful to present the frequency domain data without further processing steps and the models we have developed for healthy skin cannot be applied directly. In this work we therefore look at the impulse response functions and observe how they change during the measurement by plotting the peak to peak as a function of measurement time. We know from our previous work that a lower reflected peak and a flatter occlusion curve can indicate greater hydration.

Impulse functions are generated using a measurement of air for the reference and the Equation:

1 $$\begin{aligned} I_{sample}(t) =\mathscr {F}^{-1} \left( \frac{\mathscr {F}(E_{sample}(t))}{\mathscr {F}(E_{reference}(t))}\times DG(\omega )\right) \end{aligned}$$

where $I_{sample}(t)$ is the impulse function of the sample, $\mathscr {F}$ is the Fourier transform operator, $E_{sample}(t)$ and $E_{reference}(t)$ are the THz electric field of the sample and reference respectively. $DG(\omega )$ is a double Gaussian frequency filter used to suppress high frequency noise and is defined by:

2 $$\begin{aligned} DG(\omega )= & {} \left( e^{(\frac{-H\omega }{2})^2} - e^{(\frac{-L\omega }{2})^2} \right) e^{1j\frac{\omega }{2f_{s}}} \end{aligned}$$

3 $$\begin{aligned} H= & {} \frac{2}{\omega _{upper}} \; L = \frac{2}{\omega _{lower}} \end{aligned}$$

where $\omega _{upper}$ and $\omega _{lower}$ are the upper and lower frequency bounds, and $f_s$ is the sampling frequency. In this work, the upper and lower frequency bounds are set as 1.0*THz* and 0.1*THz*.

### Measurement stability

During a measurement, the act of pressing the quartz imaging window to the surface of the skin has an affect on it. The quartz window traps moisture inside the skin^17^ causing increased hydration in the SC, and flattening the surface of the skin. This occlusion process improves the contact between the quartz window and the skin^18^ and manifests in small changes to the impulse function.

The greatest change in the occlusion curve is in the first 5-10 seconds of the skin making contact with the quartz window and it is relatively stable by 30- 60 seconds, thus we can make meaningful comparisons between volunteers if we just measure for 60 seconds^17^. Furthermore, a longer measurement would be more strenuous for the operator, and more inconvenient and uncomfortable for volunteers. These factors would reduce the consistency in the pressure and positional stability, in turn affecting the THz measurement

**Figure 2:**
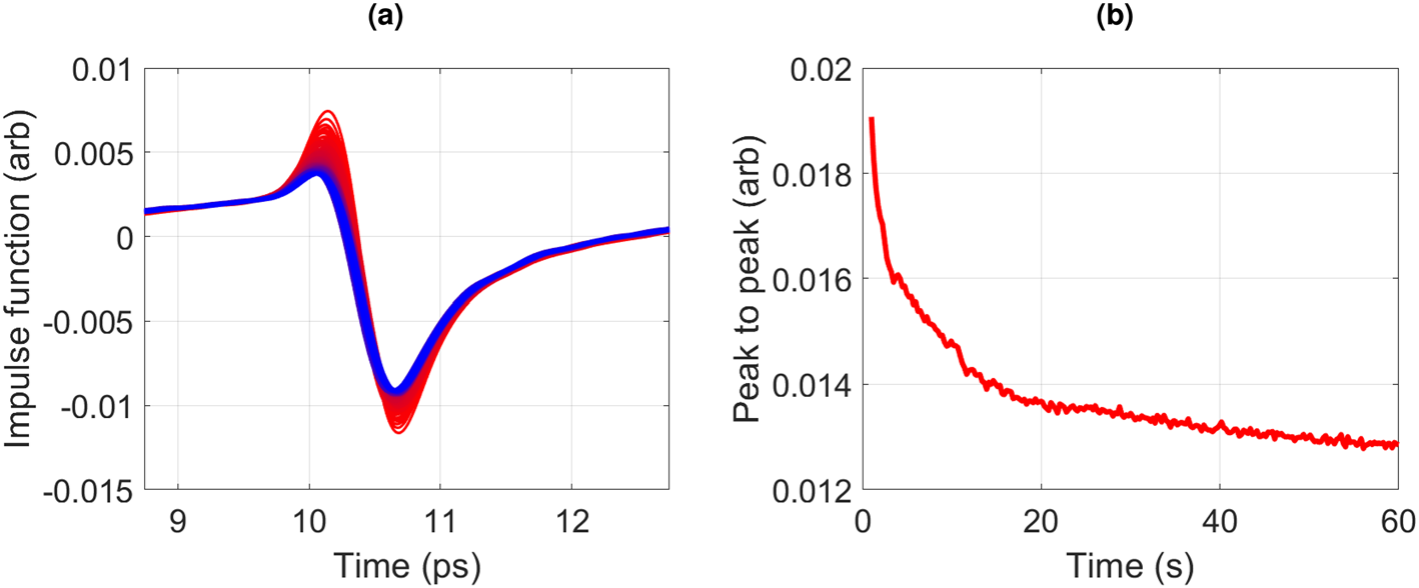
(**a**) All the impulse functions recorded during a 60 s measurement of the healthy volar forearm of a patient: where red corresponds to earlier in the measurement time. (**b**) The peak to peak of the impulse function a function of the measurement time.

Figure 2 shows the impulse functions 2a measured over 60*s* of a volar forearm of a patient, the red measurements correspond to the early seconds of the measurement and are coloured more blue as the measurement progresses. The peak to peak of the impulse function decreases over time (shown in Fig. 2b). Due to this systematic change to the signal, impulse functions taken 55*s* into the measurements are often used when making comparisons between skin areas and patients, as this gives a more stable result.

### Relative difference calculation

To make meaningful comparisons between patients, we calculate the relative difference between measurements for each participant. For example, we use Equation 4 to compare the difference in amplitude of the peak (or trough) of the psoriasis impulse function to the healthy impulse function relative to that of the healthy skin.

4 $$\begin{aligned} \text {Relative difference in trough} = \frac{A_{dry}^t - A_{healthy}^t}{A_{healthy}^t} \times 100 \end{aligned}$$

where $A_{dry}^t$ is the amplitude of the trough of a dry skin impulse function, and $A_{healthy}^t$ is the amplitude of the trough of the corresponding healthy skin impulse function. To compute the relative difference in the maximum, Equation 4 is used, with the values $A_{dry}^t$ and $A_{healthy}^t$ replaced with $A_{dry}^p$ and $A_{healthy}^p$: the peak of the dry skin impulse function and the peak of the healthy skin impulse function.

### Psoriasis

**Figure 3:**
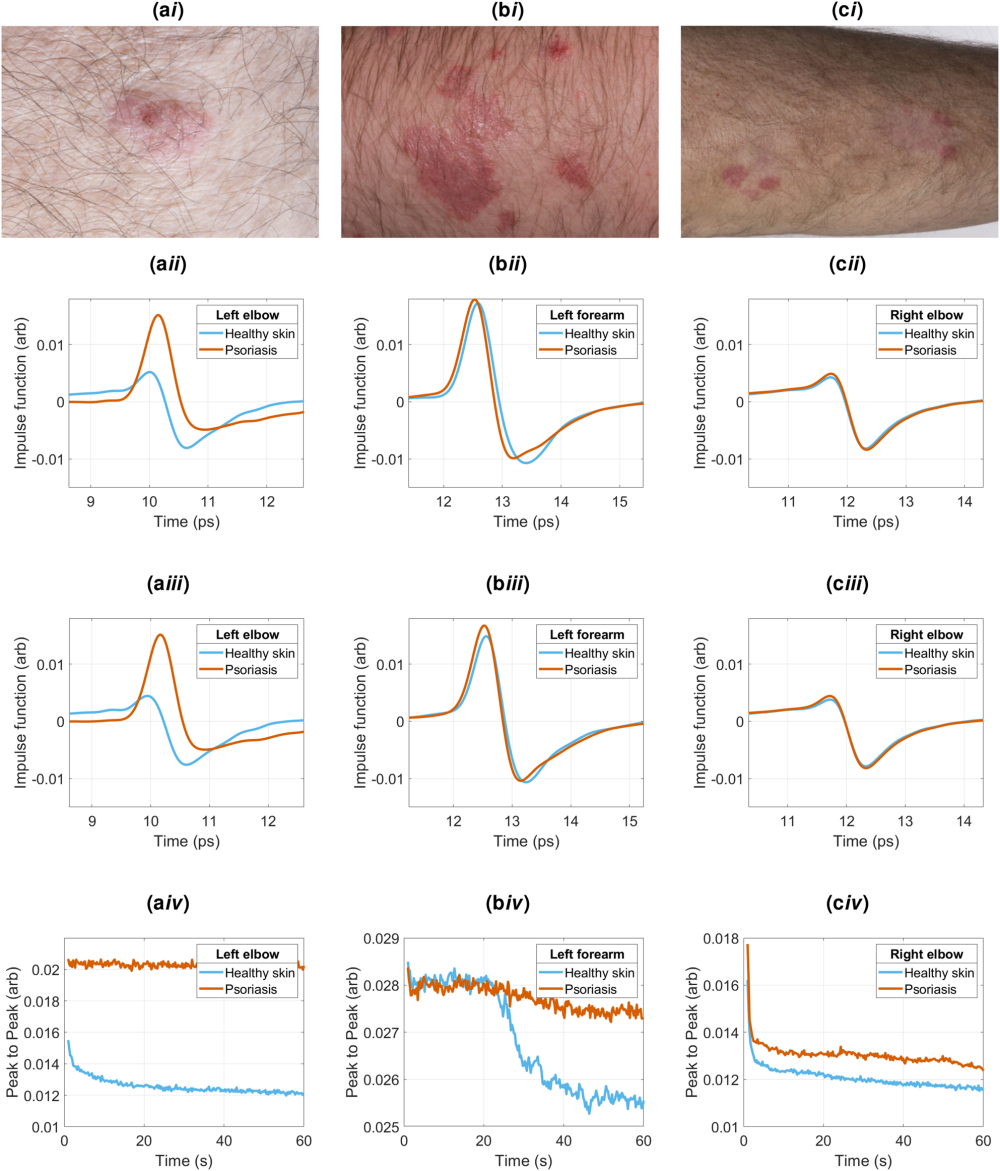
Photos of psoriasis on different patients on the (**ai**) forearm, (**bi**) left elbow and (**ci**) right elbow. Corresponding impulse function measurements at 5 sinto occlusion for the (**aii**) forearm, (**bii**) left elbow and (**cii**) right elbow. Corresponding impulse function measurements at 55 s into occlusion for the (**aiii**) forearm, (**biii**) left elbow and (**ciii**) right elbow. Corresponding occlusion curves for the (**aiv**) forearm, (**biv**) left elbow and (**civ**) right elbow.

The symptoms of psoriasis vary from person to person. Figure 3ai,bi,ci show photos of psoriasis on different patients, with the severity decreasing from left to right (Fig. 3ai–ci). The skin structure is clearly very different for each patient: Fig. 3ai shows a localised scab on the forearm, Fig. 3bi shows a thick patchy plaque on the elbow, and Fig. 3ci shows a region of much thinner psoriasis on the elbow. Each ROI was measured for 60s and we present the resulting impulse functions from the 5s and 55s time points to show how they change for different patients and skin conditions. Figure 3aii,bii,cii show the impulse functions at 5*s* corresponding to the psoriatic skin (orange) and healthy skin (blue) from the ROIs in photos Fig. 3ai,bi,ci respectively. The impulse functions measured at 55*s* are shown in Fig. 3aiii,biii,ciii. In all cases the impulse function for psoriasis has a higher peak than the healthy skin. This is typical of all the measurements made of psoriasis, and is different from what we observe for the eczema measurements (presented in the next section).

The peak to peak of the impulse functions throughout the measurement as a function of measurement time is shown in Fig. 3aiv,biv,civ. The peak to peak shown in Fig. 3bii,cii shows a steady decrease with time before settling at a fixed value, for both the healthy skin and psoriasis measurement. However, Fig. 3aiv shows a discontinuity in the peak to peak of the healthy skin after 20*s*. This is likely to be caused by the probe moving slightly during the measurement to form a better contact with the skin after 20*s*: by 50*s* the peak to peak has stabilised to the same level as the psoriasis measurement peak to peak. Thus it is useful to measure the skin for 60s to both see any trends and also be sure that the data are taken with good contact of the probe on the skin.

The impulse function shown in Fig. 3aiii has the highest peak, and the photo shows the thickest layer of psoriasis on the skin. In contrast, Fig. 3ciii has the smallest peak, and corresponds to the photo in Fig. 3ci with the thinnest layer of psoriasis. This suggests that the amplitude of the peak of the signal is related to the thickness of the psoriasis on the surface of the skin. In Fig. 6, the 0 minutes measurements, i.e. before the emollient was applied, indicate how the THz parameters of the psoriatic skin for all 24 participants were consistently different from the healthy skin. In particular, the peak of the impulse function of the psoriasis was $31 \%$ higher or more than the healthy skin for more than $75\%$ of the subjects.

### Eczema

**Figure 4:**
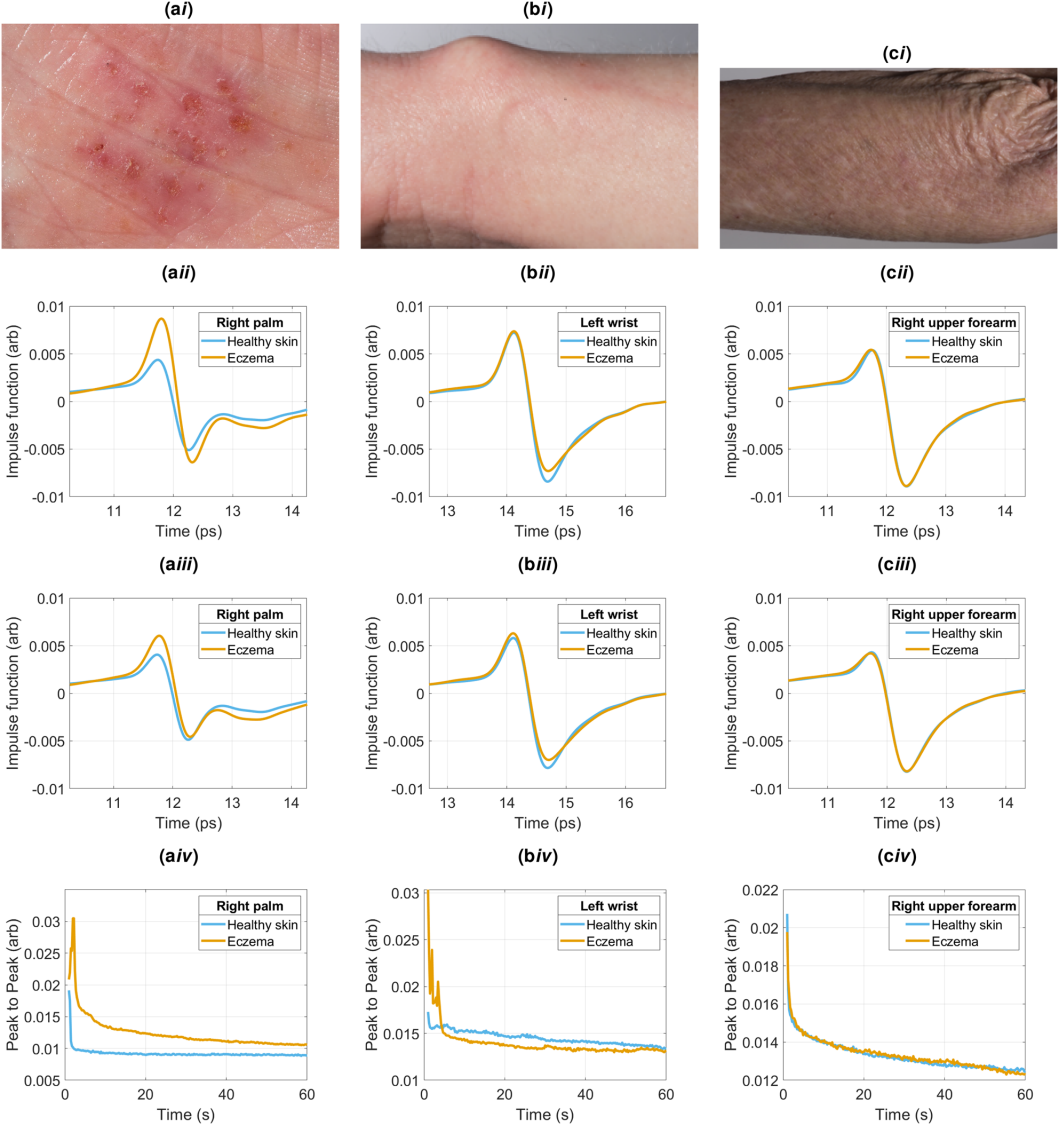
Photos of eczema on different patients on the (**ai**) right palm, (**bi**) left wrist and (**ci**) upper forearm. Corresponding impulse function measurements at 5 seconds into occlusion for the (**aii**) right palm, (**bii**) left wrist and (**cii**) upper forearm. Corresponding impulse function measurements at 55 seconds into occlusion for the (**aiii**) right palm, (**biii**) left wrist and (**ciii**) upper forearm. Corresponding occlusion curves for the (**aiv**) right palm, (**biv**) left wrist and (**civ**) upper forearm.

The impulse function measured from eczema also varies depending on both the region measured and the severity of the eczema. Figure 4 shows photos of 3 different patients (Fig. 4ai,ci,bi) with the corresponding eczema and healthy skin measurements after 5*s* below (Fig. 4aii,cii,bii), followed by the measurements after 55*s* (Fig. 4aiii,ciii,biii). The peak to peak of the impulse functions throughout the measurement as a function of measurement time is shown in Fig. 4aiv,civ,biv).

Figure 4aiii shows a measurement of a palm after 55*s*, with both the healthy and eczema measurements showing a second trough. This is due to the thick outer palmoplantar layer of skin on the palm. It is also clear to see that the peak of the signal is larger for the eczema measurement. Figure 4biii shows a wrist measurement with no second trough as there is no thick outer layer in that region of the body. The peak of the eczema measurement is also higher than the healthy measurement in Fig. 4biii, but not to the same extent as the palm measurement. The photos in Fig. 4ai,bi show that the eczema measured in Fig. 4aiii has a less obvious scabbed dry layer on the outside compared to that shown in Fig. 4ai. The thicker rough eczema layers on the surface of the skin lead to a larger peak in the signal and this is consistent with the observations about psoriasis measurements. Noteworthy, for this most severe case of ezcema presented on the palm, the high peak measured at 5s decreases significantly more than the healthy palm during occlusion: the impulse functions are significantly closer together in Fig. 4aiii than they are in Fig. 4aii. This is due to the occlusion process re-hydrating the dry SC on the palm such that it starts to get closer to the healthy skin response.

Figure 4ciii shows a measurement of a forearm. It can be seen Fig. 4ci that the skin appears dry in both the eczema region and the surrounding areas. In this measurement of the eczema and healthy skin, the two regions can not be distinguished by their impulse functions alone: this is not uncommon with the measurements of eczema patients, especially when the eczema is under control, and could be due to usage of moisturisers. In Figure 7, the 0 minutes measurements (before the emollient was applied) indicate how the THz parameters of the eczema skin for all 13 participants differed from the healthy skin. It is only for the peak to peak parameter that there was a statistically significant difference across most of the participants: the peak to peak of the impulse function of the eczema was at least $11 \%$ greater than the healthy skin for more than 75% of the subjects. This is likely to be due to the variation in severity and location of the eczema.

### Impact of emollient

**Figure 5:**
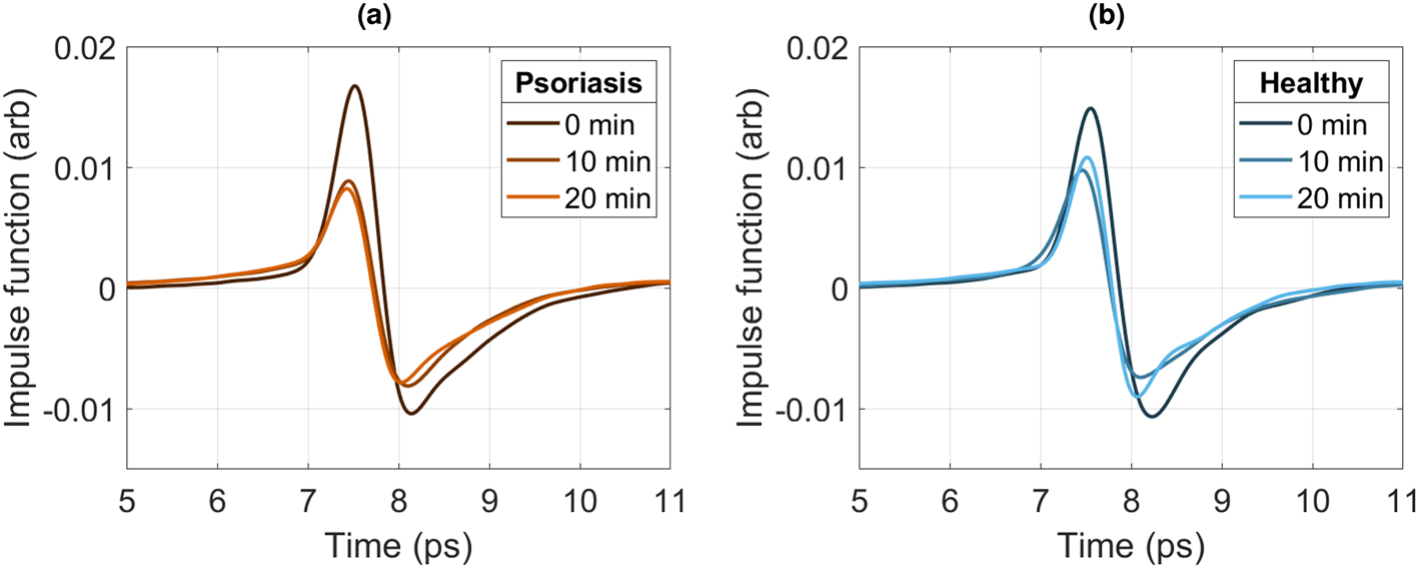
The impulse function of psoriatic skin shown in Fig. 3bi, (**a**) and after 55*s* of measurement, and the corresponding healthy skin (**b**) 0 minutes, 10 minutes, and 20 minutes after the application of emollient.

Emollient was applied to both the psoriatic skin and and healthy skin, and both areas were measured again after 10 and 20 minutes. It is worth noting the limitation that all patients applied their own moisturiser as per the protocol (so as not interfere with their treatment) but this meant that we could not apply the same moisturiser product nor measure the same volume of product to all participants and this may account for some outlying data points. The resultant impulse functions for the patient photographed in Fig. 3bi are shown in Fig. 5. The peak of the impulse function decreases after 10 minutes in both the healthy and psoriasis skin, and then after 20 minutes, the psoriasis skin peak further decreases in amplitude. The trough of the psoriasis and healthy skin impulse function also decreases after 10 minutes. After 20 minutes the psoriasis impulse function decreases further than the healthy skin impulse function. This could be because the psoriatic plaque soaks up more of the moisturiser and less of it sinks through to the epidermis. In the healthy skin, the SC will absorb the moisturiser and interact with the epidermis to restore its hydration level back to normal.

The trend shown in Fig. 5 is generally found in psoriatic skin. To better understand the effects at play when applying moisturiser, we now also consider the changes in the maximum amplitude (peak) and minimum amplitude (trough) separately and compute these values for all patients using Rquation (4).

**Figure 6:**
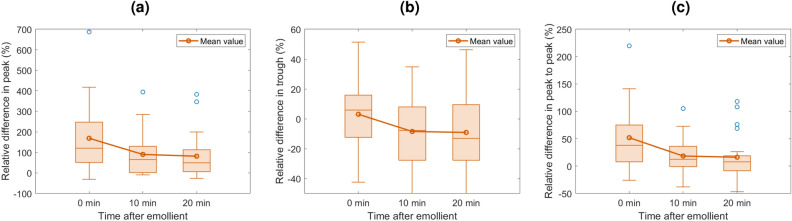
Box and whisker plots showing the relative change in the peak (**a**), trough (**b**) and peak to peak (**c**), of impulse functions of psoriatic skin as a function of time after emollient application. Calculated using Eq. 4. All 24 psoriasis patients’ datasets were included. Note the vertical scales on each plot have different ranges to better illustrate the results.

**Figure 7:**
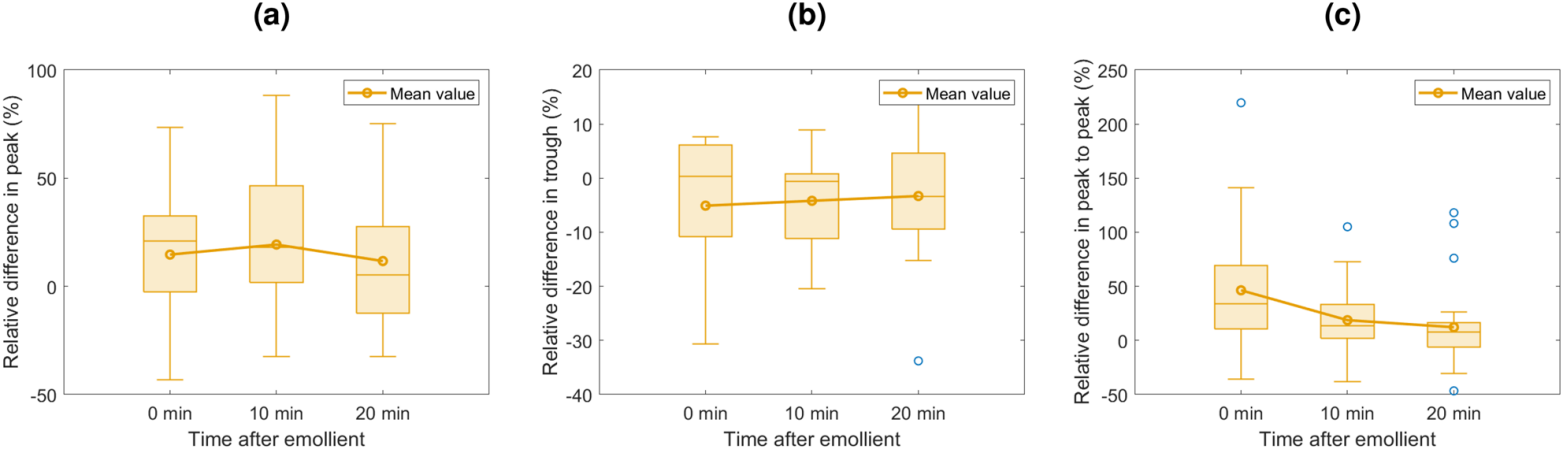
Box and whisker plots showing the relative change in the peak (**a**), trough (**b**) ans peak to peak (**c**), of the impulse functions for eczema affected skin as a function of time after emollient application. Calculated using Eq. 4. All 14 eczema patients’ datasets were included. Note the vertical scales on each plot have different ranges to better illustrate the results.

The relative changes in the peak, trough and peak to peak as a function of time after emollient are shown in Fig. 6a–c for psoriatic skin, and Fig. 7a–c for eczematic skin.

Figure 6a shows that despite some outliers, there is a statistically significant trend of the peak of the psoriasis impulse function having a larger signal amplitude before emollient application, and then that difference shrinks as the emollient soaks into the skin. Figure 6b shows a weak trend of a larger trough in the impulse function of psoriasis compared to healthy skin, but after emollient is applied the trough for the impulse function becomes smaller than that of healthy skin. Figure 6c shows that in general the peak to peak of the impulse function from psoriatic skin is larger than for healthy skin, and decreases after emollient is applied, however this is a weaker trend than that of just the peak. Thus we can deduce that the dominant effect is on the peak and this physically corresponds to the surface information and is most likely due to the psoriatic plaques becoming smoother when moisturised.

The relative changes to the peak and trough for the eczema patients (Fig. 7a,b) do not have significant trends with the application of moisturiser, but interestingly, the peak to peak, Fig. 7c does show a trend. It is noteworthy that for the patients with more severe eczema, we are able to see a clearer distinction. An example is shown in Fig. 4ai where the skin is scabbed and rough, therefore the peak of the signal is larger, and so is the trough. However in general the clearest indicator of eczematic skin is the peak to peak of the signal.

It is likely that we do not see as clear trends in the eczema data as in the psoriasis data because there is a greater variation in severity and symptoms of those patients measured with eczema. Furthermore, from the diagrams in Fig. 1 it can be seen that while psoriatic skin has a thick layer on the top surface, eczema does not have a fundamentally different structure. This means we would expect the impulse functions of healthy and eczematic skin to be more similar to each other compared to healthy and psoriatic skin. When the skin is behaving more closely to that of healthy skin, we are then able to use our previous analysis techniques from^12^ to determine the hydration and thickness of the SC and this could be used to quantitatively monitor patients and/or give them positive feedback alongside their treatment plan.

## Discussion

In this study a range of anatomical regions were measured for patients with psoriasis or eczema. We have seen that THz light is clearly sensitive to changes in skin caused by skin conditions and also by the application of moisturiser. In our previous study of healthy skin, we were able to fit the data to a multi-layer model and extract the SC thickness and hydration, but it is not so straightforward to do this for dry skin. Here we have therefore reported our observations from applying the basic analysis technique of deconvolution and calculation of relative change coefficients.

When emollient is applied to psoriatic skin, it becomes more similar to the healthy skin. In particular it softens the top surface, reducing the microscopic air gaps between the quartz imaging window of the measurement probe and the skin. This change in the skin surface accounts for the change in the peak of the signal. The later parts of the impulse function can be considered as the result of reflections from deeper layers in the skin, based on their hydration. The clearest manifestation of this is the trough of the signal, for example we can see a change to the trough of the signals in Fig. (5) when emollient is applied. The expected result is that the trough would decrease in amplitude as the skin hydrates over time, and this can be seen in the difference between the trough of the signal measured at 0 minutes compared to that at 10 minutes. However the change is more subtle between 10 and 20 minutes, and the trough even becomes larger for many participants between the 10 and 20 minute measurement, possibly an indication of the skin trying to start recovering to its initial hydration state. This variety in observations at this time point could be due to participants applying different amounts of moisturiser as well as different mechanisms being at play and is indicative of the complex nature of skin and the need to investigate this more comprehensively. It is more difficult to see trends in the eczema data, but this is due to multiple factors including the variation in severity affecting the skin in different ways; the variance in moisturiser type and volume applied; and the location of the eczema.

The reflection of THz light from within the skin is dependent on its hydration profile as this in turn affects its refractive index^3,11,12^. The refractive index is frequency dependent, and from Fresnel theory, the contrast increases with frequency. However, the signal to noise ratio of the THz system decreases as the frequency increases and this is compounded by the higher frequencies being attenuated more by the skin. When generating impulse functions using Equation 1, the double Gaussian filter described in Equation 2 heavily attenuates the high frequency components and over compensates for the higher frequency noise in the handheld THz set up. Thus, it will be beneficial to devise analysis techniques that extend the useful frequency range of the set up so as to gain a better contrast between the THz responses of healthy and dry skin. This is the subject of further investigation.

In summary, our key findings from this study are 1) the THz signal for psoriatic skin is statistically significantly different from the corresponding normal skin, with the peak of the impulse function being the best at identifying this and with thicker psoriasis corresponding to a larger peak. 2) The THz response of eczematic skin is different from psoriatic skin and the peak to peak of the impulse function is our best parameter so far for distinguishing it from surrounding healthy skin. This pilot study is the first of its kind and has generated a wealth of data. We are still investigating what will be the best analysis techniques to determine the most useful parameters for patients and clinicians, and this will be reported in a follow up paper.

## Methods

We have conducted the first in vivo pilot study measuring patients with dry skin conditions using THz sensing. The study has been carried out at the University Hospital Coventry and Warwickshire (UHCW). Patients with psoriasis and eczema were measured using a handheld THz probe designed in house^12^.

Patients were asked a range of questions about their health, and were measured with THz before and after emollient application to show the sensitivity of the THz probe to dynamic changes induced in the skin. Appropriate informed consent was taken from each patient. Several patients agreed to have photos taken of their conditions for comparison.

Ethical approval was awarded by the Health Research Authority (HRA) and Health and Care Research Wales (HCRW) for The SINATRA study: SkIN hydrAtion evaluation with TeRAhertz scanning, with IRAS project ID: 270335. All research was performed in accordance with relevant guidelines and regulations, including in accordance with the Declaration of Helsinki. Informed consent was obtained from all participants.

### Handheld set up

**Figure 8:**
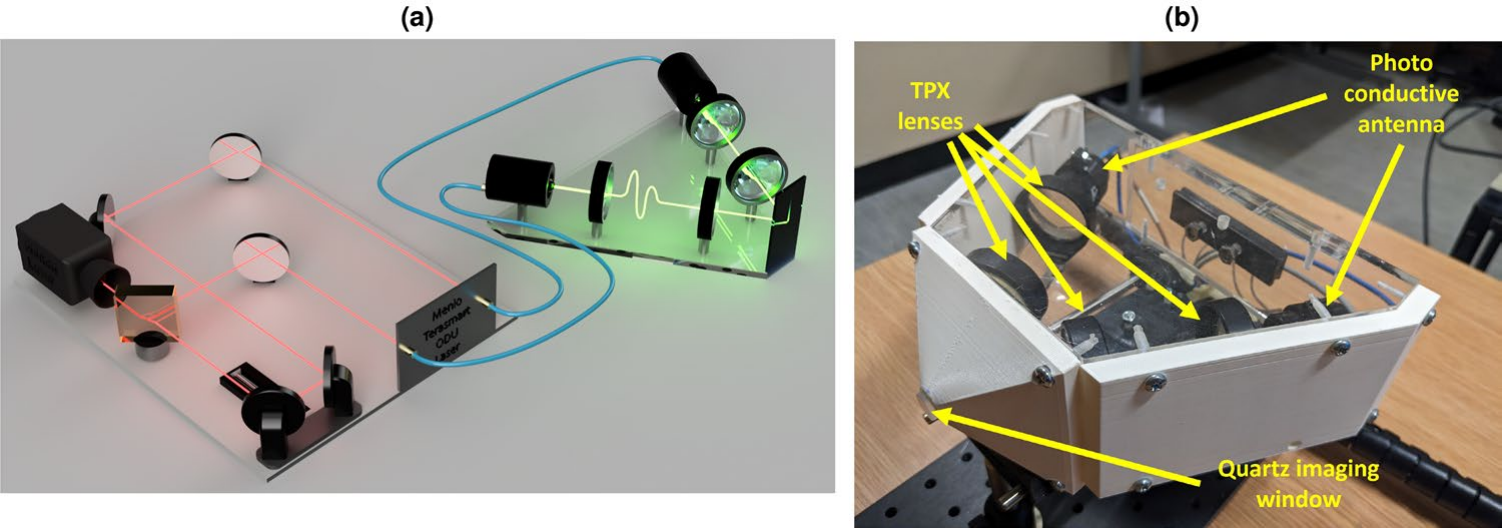
(**a**) shows a diagram of a typical THz TDS reflection system: a femtosecond laser is split by a beamsplitter. One part of the beam is sent to an photoconductive antenna serving as an emitter and the other to a photoconductive antenna serving as a detector. The path length to the detector is altered by changing the length inside the optical delay unit (ODU). The THz light from the emitter is focused by a pair of TPX lenses onto to outer face of a quartz sampling window. The resulting reflection is focused by a second pair of lenses into the detector. The generated electric signal is processed by a lock-in amplifier and a computer system inside the Menlo Terasmart. This mechanism has been compacted into the handheld Skinometer device shown in (**b**). The diagram in Fig. 8 was produced in AutoDesk Fusion 2024 (https://www.autodesk.co.uk/products/fusion-360).

**Figure 9:**
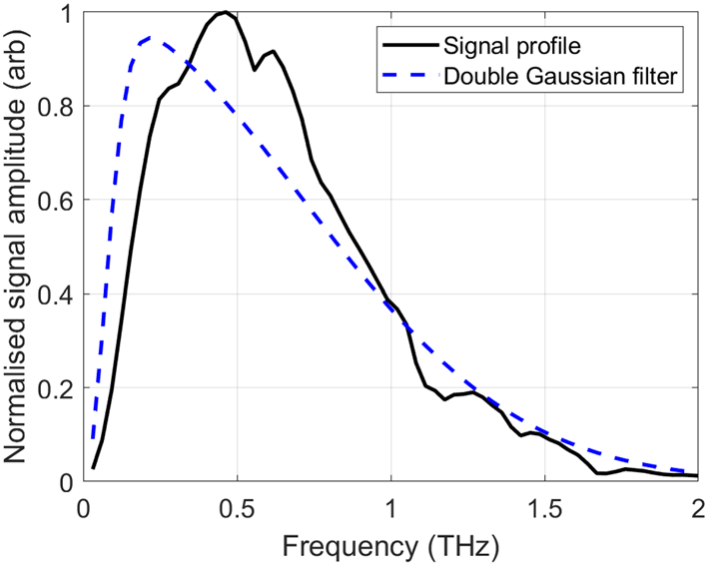
A typical signal profile of the Skinometer handheld probe shown in Fig. 8b, together with the profile of the double Gaussian filter used to generate the impulse functions as described in Eq. 1.

Due to the limited penetration depth of THz in skin (about $100\mu m$), in vivo measurements must be made in reflection geometry. An imaging window is needed to ensure good alignment of the sample with the THz focusing optics. Reflection THz TDS systems have been widely used in other in vivo studies^4,19^ but these have typically been bench top set ups^17^. However, in this work we use a handheld reflection device: the Skinometer, as demonstrated in^12^. The Skinometer features a small imaging window that can be pressed on difficult-to-reach areas at unusual angles, such as the nose, the jaw, and the lower back. A brief schematic diagram of a THz TDS reflection system is shown in Fig. 8a. A Menlo Terasmart is used to pump a GaAs photoconductive antenna, the resultant admitted terahertz is collimated by a TPX lens and then focused onto the sample, through a quartz imaging window, by a second lens. The angle of incidence onto the sample is 30$^{\circ }$. The reflected signal is collimated by a TPX lens and finally refocused into a second photoconductive antenna which serves as a detector. To construct the Skinometer, the photoconductive antenna, the TPX lenses and the quartz imaging window have been packaged into a lightweight, mobile device. This is shown in Fig. 8b. A pressure sensor has been incorporated into the handle of the device to ensure that the imaging window is being applied with even pressure as this has been shown to impact in vivo measurements^17^. The signal profile of the handheld device is shown in Fig. 9, along with the double Gaussian used to filter data for generating impulse functions, as described in Equation 1. The values used for the upper and lower frequency bounds of the double Gaussian are 1.0 THz and 0.1 THz.

### Measurement protocol

The measurements were carried out at University Hospital of Coventry and Warwickshire (UHCW). Patients with dry skin conditions found in areas accessible by the Skinometer scanner were recruited by staff at the hospital. Appropriate consent was taken from each patient.

On arrival each volunteer was asked to expose the area with the dry skin condition and a corresponding healthy area on the other side of their body so the skin can acclimatise to conditions of the room. They were then asked to fill a questionnaire containing questions regarding their lifestyle, age, and general health. A measurement was then taken using handheld probe of their volar forearm, the region with the skin condition, and the corresponding healthy region. Emollient was then applied by the volunteer to the skin condition region and the healthy region, these areas were then measured again 10 minutes after emollient application and 20 minutes after emollient application.

In the case that multiple areas of dry skin were available, the area chosen was one that is both easier to measure (i.e. not on a bone or such) and has a convenient corresponding healthy region on the other side of the body. For example: if a patch of psoriasis is measured on the left shoulder then the healthy region will be the same area on the right shoulder. Sometimes the dry skin condition will be so widespread that it is not possible to make a measurement of a corresponding healthy region, in that case the nearest area of healthy skin was chosen.

Volunteers were also asked to have photos taken of the regions measured. These photos were taken by the medical photography team at UHCW.

## Data Availability

The data presented in this article are publicly available on Figshare at 10.6084/m9.figshare.25827406.
